# Feeding Next‐Generation Nanomedicines to Europe: Regulatory and Quality Challenges

**DOI:** 10.1002/adhm.202301956

**Published:** 2023-09-17

**Authors:** Umberto M. Musazzi, Silvia Franzè, Fabrizio Condorelli, Paola Minghetti, Paolo Caliceti

**Affiliations:** ^1^ Department of Pharmaceutical Sciences Università degli Studi di Milano via G. Colombo Milan 71‐20133 Italy; ^2^ Department of Pharmaceutical Sciences Università degli Studi del Piemonte Orientale Largo Donegani Novara 2‐28100 Italy; ^3^ Department of Pharmaceutical and Pharmacological Sciences University of Padova via F. Marzolo Padova 5‐35131 Italy

**Keywords:** nanomaterials, nanomedicines, physicochemical characterization, quality attribute, regulatory science

## Abstract

New and innovative nanomedicines have been developed and marketed over the past half‐century, revolutionizing the prognosis of many human diseases. Although a univocal regulatory definition is not yet available worldwide, the term “nanomedicines” generally identifies medicinal products that use nanotechnology in their design or production. Due to the intrinsic high structural complexity of these products, the scientific and regulatory communities are reflecting on how to revise the regulatory framework to provide a more appropriate benefit/risk balance to authorize them on the market, considering the impact of their peculiar physicochemical features in the evaluation of efficacy and safety patterns. Herein, a critical perspective is provided on the current open issues regarding regulatory qualification and physicochemical characterization of nanosystems considering the current European regulatory framework on nanomedicine products. Practicable paths for improving their quality assurance and predicting their fate in vivo are also argued. Strengthening the multilevel alliance among academic institutions, industrial stakeholders, and regulatory authorities seems strategic to support innovation by standard approaches (e.g., qualification, characterization, risk assessment), and to expand current knowledge, also benefiting from the new opportunities offered by artificial intelligence and digitization in predictive modelling of the impact of nanomedicine characteristics on their fate in vivo.

## Graphical abstract

Nanomedicines are revolutionizing the prognosis of many human diseases but their peculiar physicochemical properties are raising new challenges in regulatory approval. Current open issues regarding regulatory qualification and physicochemical characterization of nanosystems are critically discussed, considering the current European regulatory framework on nanomedicine products. Practicable paths for improving their quality assurance and predicting their fate in vivo are also argued.

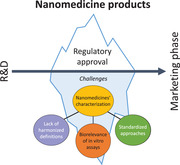

## 1 Introduction

Since when liposomes were first observed by Alec Bangham in the 1960s, significant advances in research have enabled the exploitation of nanomaterials and nanotechnologies for biomedical applications^[^ ^1^ ^]^ and for marketing innovative nanomedicines with tailored biopharmaceutical and biological profiles.^[^ ^2^ ^]^

Although a univocal definition is still not available worldwide, the term “nanomedicines” (or “nanomedicine products”) is generally used, from a scientific point of view, to identify medicinal products that exploit nanotechnologies in their design or production and possess physical properties that comply the nanomaterial definition adapted to pharmaceuticals. Consequently, nanomedicines' development, manufacturing, and marketing fall under the provisions of the existing regulatory frameworks on medicinal products in both the European Union (EU) and the United States (US).

Unlike conventional formulations (e.g., tablets, syrups), which are designed to release the drug payload before passage through biomembranes and which do not affect transport mechanisms, nanomedicine products play a crucial role in drug transport across the body compartments, dictating their fate. Therefore, their absorption, distribution, metabolism, excretion, toxicity profile is determined by the nanosystem rather than the drug. This is due to the peculiar physicochemical properties of these systems, whose performance is strongly determined by their physicochemical properties (e.g., size, polydispersity, shape, architecture, density, surface features) rather than by the overall mass properties. Indeed, the nanoscale size allows for these systems to penetrate and diffuse into and through tissues, while the large surface area is responsible for interactions with biostructures such as proteins and cells. Consequently, when introduced into the body, independently from their medical purpose, nanosystems may entail toxicological risks related to unknown, unpredictable, or unwanted distribution in tissues and interactions with biological constituents.^[^ ^1^ ^]^

Notwithstanding the opportunities offered by nanotechnology in the development of innovative drug delivery systems, there are still several gaps between scientific knowledge and the current regulatory framework.^[^ ^3^, ^4^ ^]^ Since slight differences in the physicochemical properties of raw materials or manufacturing processes can have a strong impact on the quality profile and in vivo behavior of nanomedicines, it is more complex to predict their fate as well as toxicological or therapeutic effects despite what happens with conventional medicinal products. Accordingly, at the regulatory level, nanomedicines were included in the broad class of nonbiological complex drugs (NBCDs), together with other products having a high intrinsic complexity, supramolecular structures, and compositions, which cannot allow for full physiochemical characterization.^[^ ^5^ ^]^ In light of this, for registration purposes, the in‐depth physicochemical characterization and behavior profiling of nanopharmaceuticals inside body compartments are mandatory steps for adequate pharmaceutical development that passes through the full understanding of what correlates their critical quality pattern to their efficacy and safety features.^[^ ^1^ ^]^ Such peculiarities have a relevant impact not only on the first‐in‐human approval of nanomedicines but also on the assessment of the pharmaceutical equivalence after postmarketing modifications or on the evaluation of therapeutic equivalence toward nanomedicine originators necessary for the registration of copies/follow‐on products.

However, the scientific and regulatory debate on how regulatory agencies assess the benefit/risk balance of nanomedicines, in terms of efficacy and safety patterns, for granting a marketing authorization is far to reach an international consensus. Like other medicines with a high intrinsic complexity and/or indications of unmet medical needs, the key point is to reach an equilibrium between the regulatory commitment to preserve public health by avoiding the marketing of ineffective or unsafe products and the need to support pharmaceutical innovation through regulatory pathways that allow for accelerated authorization, especially in the case of unmet medical needs.^[^ ^5^, ^6^ ^]^

Both the European Medicines Agency (EMA) and the U.S. Food and Drug Administration (FDA) have released several guidelines and reflection papers to support pharmaceutical stakeholders in assessing properly the quality, efficacy, and safety profiles of such products.^[^ ^3^, ^7^, ^8^ ^]^ To take the first move to reach the established regulatory requisites for nanopharmaceuticals, several new methods, techniques, and analytical protocols have been developed to characterize the nanoscale nature of nanomaterials and nanomedicines.^[^ ^9^, ^10^, ^11^ ^]^ In parallel, the advances in artificial intelligence (AI)/machine learning (ML) applications are opening new chances and challenges in all preauthorization steps of medicinal product development, as documented in a recent EMA reflection paper.^[^ ^12^ ^]^ For example, AI/ML models and digitalization are revolutionizing the toxicological assessment of nanomedicines.^[^ ^13^, ^14^ ^]^ However, the worldwide lack of harmonized standards in the characterization of nanosystem structures and their performance is driving the development of accurate and reliable AI/ML models to predict their interaction with biological constituents and, therefore, their toxicological effects.

**Figure 1:**
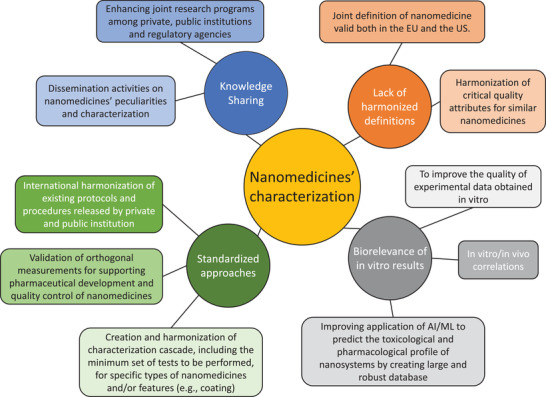
Challenges (round shape) and solutions (rectangle‐shape) in physicochemical characterization of nanomedicines

Here we decided to analyze and discuss what we considered to be among the most pressing critical issues still on the ground in terms of qualification, physicochemical characterization of nanosystems. As well, the most affordable paths, in our opinion, to improve both their quality assurance and the prediction of their fate in vivo were argued, following the current European regulatory framework on nanomedicine products (**Figure** **1**). For a general overview of the regulatory challenges to the use of nanomaterials and/or nanotechnologies use in medical devices, cosmetics, and food, you can refer to the reviews already published on these topics.^[^ ^3^, ^15^, ^16^, ^17^ ^]^

## 2 Regulatory Qualification of Nanomedicine Products

The class of nanomedicines appears a very heterogeneous cluster of nanotechnological structures. It includes different colloidal systems, so‐called nanosystems, that through drug encapsulation or chemical or physical association, can improve drug stability, apparent solubility and distribution in body and cells, favor the targeting of tissues and/or organs, and not least, work as gene vectors and vaccine adjuvants.^[^ ^18^ ^]^ Therefore, one of the critical issues in the approval processes of nanomedicines is related to the broad definition of nanotechnology‐based systems (i.e., nanosystems) for medical purposes that identifies the structural and physicochemical features of these products concerning their use and fate.

The absence of a consensus on defining nanomedicines has generated a plethora of terms adapted to identify related items (e.g., nanomaterials, nanomedicines, nanopharmaceuticals, nanoparticles, and nanosystems), which has expanded the regulatory uncertainty in the field. For the sake of clarity, the terms “nanomedicines” or “nanomedicine products” have been used throughout the paper for indicating nanosized systems developed for therapeutic/diagnostic purposes in line with the EMA reflection paper of 2006 (**Table** **1**). Similarly, the term “nanomaterials” is referring to solid nanosized particles, regardless they are designed for pharmaceutical applications or not‐pharmaceutical ones (e.g., cosmetics, foods, tattoo inks), in agreement with the definitions of the European Commission (EC) of 2011 and 2022 (Table 1).^[^ ^3^, ^19^ ^]^ Instead, the term “nanosystems” is used to generally identify nanotechnology‐based delivery systems for bioactive molecules.

**Table 1:** Definitions relevant to the regulatory framework on nanomaterials and nanomedicines.

Sources	Terms	Definitions/points‐to‐considered	Ref.
European commission (2011)	Nanomaterial	1. “Nanomaterial” means a natural, incidental, or manufactured material containing particles, in an unbound state or as an aggregate or as an agglomerate and where, for 50% or more of the particles in the number size distribution, one or more external dimensions is in the size range 1–100 nm. In specific cases and where warranted by concerns for the environment, health, safety, or competitiveness the number size distribution threshold of 50% may be replaced by a threshold between 1% and 50%. 2. By derogation from point 1, fullerenes, graphene flakes, and single wall carbon nanotubes with one or more external dimensions below 1 nm should be considered as nanomaterials.	[20]
European Commission (2022)	Nanomaterial (currently in force)	“Nanomaterial” means a natural, incidental, or manufactured material consisting of solid particles that are present, either on their own or as identifiable constituent particles in aggregates or agglomerates, and where 50% or more of these particles in the number‐based size distribution fulfil at least one of the following conditions: a) one or more external dimensions of the particle are in the size range 1–100 nm; b) the particle has an elongated shape, such as a rod, fiber, or tube, where two external dimensions are smaller than 1 nm and the other dimension is larger than 100 nm; c) the particle has a plate‐like shape, where one external dimension is smaller than 1 nm and the other dimensions are larger than 100 nm. In the determination of the particle number‐based size distribution, particles with at least two orthogonal external dimensions larger than 100 µm need not be considered.However, a material with a specific surface area by volume of <6 m^2^ cm^−3^ shall not be considered a nanomaterial.”	[19]
EMA (2006)	Nanotechnology	Nanotechnology is defined as the production and application of structures, devices, and systems by controlling the shape and size of materials at nanometer scale. The nanometer scale ranges from the atomic level at around 0.2 nm (2 Å) up to around 100 nm.	[24]
EMA (2006)	Nanotechnology (updated)	The use of tiny structures less than 1000 nm across, which are designed to have specific properties.	[25]
EMA (2006)	Nanomedicine	Nanomedicine is defined as the application of nanotechnology in view of making a medical diagnosis or treating or preventing diseases. It exploits the improved and often novel physical, chemical, and biological properties of materials at nanometer scale.	[24]
FDA (2014)	Nanotechnology	When considering whether an FDA‐regulated product involves the application of nanotechnology, FDA will ask: Whether a material or end product is engineered to have at least one external dimension, or an internal or surface structure, in the nanoscale range (≈1–100 nm); Whether a material or end product is engineered to exhibit properties or phenomena, including physical or chemical properties or biological effects, that are attributable to its dimension(s), even if these dimensions fall outside the nanoscale range, up to 1 µm (1000 nm).	[26]
NBCD Working Group (2014)	Nonbiological complex drugs	Medicinal products, not being biological medicines, where the active substance is not a homomolecular structure, but consists of different (closely) related and often nanoparticulate structures that cannot be isolated and fully quantitated, characterized, and/or described by physicochemical analytical means	[28]

The broad definition of “nanomaterials” released by the EC in 2011 has been improperly used to classify nanosystems or engineered macromolecules designed and developed for biopharmaceutical purposes.^[^ ^20^ ^]^ Indeed, such a definition is a first attempt to guide the nanotechnology innovations and existing nanomaterials under a regulatory umbrella able to ensure public health, regardless of their field of application (e.g., consumer goods, healthcare products, semi‐finished products) or material type (i.e., natural, incidental, or manufactured). The definition has been integrated into product‐specific European regulations and directives (e.g., foods, cosmetics, medical devices),^[^ ^21^, ^22^, ^23^ ^]^ and it is enriched by the provisions needed to accurately assess the benefit/risk balance of nanomaterials use, based on the peculiarity of each product class. Nevertheless, the heterogeneity of product‐specific requirements for nanomaterials has led to misleading interpretations and difficulties in classifying nanomaterials' borderline components, structures, and systems.^[^ ^3^ ^]^ This makes it difficult for researchers and manufacturers to properly comply with the benefit/risk balance of borderline nanotechnology‐based products, due to the provisions and characterization imposed by different regulatory frameworks. In particular, this difficulty emerges significantly in the early stages of the research and development (R&D) process of a nanotechnology‐based product, where the final market application (e.g., therapeutic, or cosmetic indications) may not be yet well defined. In 2022, the EC revised the definition to overcome the criticisms of the nanotechnology field,^[^ ^19^ ^]^ leading to a more specific and detailed version that is currently in force (Table 1). The revised version clarifies that, in the European Union, the use of the term “nanomaterial” should be restricted to a solid piece of matter that fulfils at least one of the detailed conditions reported in the definition. Single molecules are not considered particles by the EC. Therefore, nonsolid structures and macromolecules are explicitly excluded by the definition.

Despite the relevant improvement made in the latest version, the EC definition remains relatively powerless in qualifying all possible nanotechnological applications in the biomedical sector, especially in the field of pharmaceutical sciences. Most of the nanoscale systems investigated in the R&D of medicinal products are still not qualifiable as nanomaterials. Among the medicinal products on the market, only iron‐based nanocolloidal products and other metallic‐core nanoparticles fulfil the definition. Conversely, liposomes, micellar systems, protein‐based nanoparticles, and other nanosystems designed for delivering and/or targeting active ingredients to living beings are not nanomaterials based on the EC definition.

Both versions of the EC definition focused more on qualifying materials, which could impact consumer safety due to their nanoscale range, rather than identifying systems that could radically change their biopharmaceutical properties as compared to the bulk material. For instance, a specification of “≥50%” of particles in the number‐based size distribution is a valid criterion for identifying material for which the nanoscale‐particle population may be toxicologically relevant. Conversely, this parameter is too broad for pharmaceutical applications, where formulation efforts are concentrated on obtaining nanosystems in which the narrower the size distribution, the more reproducible the technological and biopharmaceutical performances.

In light of this, both EMA and FDA released definitions more centered on the qualification of the innovative biopharmaceutical properties of nanosystems rather than on their nanoscale dimensions (Table 1). Nanomedicines were defined by the EMA in the reflection paper issued in 2006.^[^ ^24^ ^]^ Although the dimensional range remained relevant for qualifying nanomedicines (1–100 nm), it can be enlarged up to 1000 nm if the system is designed to have “specific properties”, which cannot be obtained by using micro‐ and macrosystems.^[^ ^25^ ^]^ Such a regulatory approach is shared with FDA, as reported in the guidance released in 2014 (Table 1).^[^ ^26^ ^]^ The widening of the size limit range for nanosystems by medicines regulatory authorities is particularly relevant for so‐called nanocrystals. In fact, unlike what happens for other formulation strategies, nanocrystals can be qualified as nanomedicine products even if larger than the 1–100 nm range, due to the significant change in the biopharmaceutical properties (e.g., drug apparent solubility) they bring to the drug.^[^ ^27^ ^]^

As highlighted by the EMA and the FDA, nanomedicines cover interdisciplinary areas and, thus, require a multidisciplinary approach and evaluation. As already stated in the Introduction, at the regulatory level, all nanomedicines are traditionally included in the broader class of NBCDs, as defined by the NBCD Working Group (Table 1).^[^ ^28^ ^]^ This appear correct although, for most of nanomedicines, the intrinsic complexity is attributable to the nanocarrier and not to the active substance itself. However, the reverse is not true: not all nanoscale NBCDs should be classified as nanomedicine products. For example, glatiramoids, complex mixtures of peptide copolymers, are NBCDs, but they do not comply with the EC definition of nanomaterials. Although some evidence suggests that they can form nanoscale 3D structures in vivo,^[^ ^29^ ^]^ they do not qualify as nanotechnologies, even based on the FDA definition, as no clear and univocal evidence have demonstrated that their mechanism of action is due to the presence of such nanostructures.^[^ ^30^, ^31^ ^]^

The proper classification of nanomedicine products affects the development toward marketing authorization of a first‐in‐human product, the management of its postmarketing modifications as well as the development of therapeutically equivalent copies at the time of patent expiration of the reference nanomedicine.^[^ ^32^, ^33^, ^34^ ^]^ Indeed, due to the inherent complexity, the equivalence of NBCDs and nanomedicine products cannot be assessed, as it occurs for generics, based on pharmaceutical equivalence and bioequivalence.^[^ ^3^, ^5^ ^]^ On the one hand, there is still the need to expand the arsenal of validated methodologies for the characterization of nanomedicines.^[^ ^35^, ^36^ ^]^ On the other hand, as far as bioequivalence is concerned, the measurement of the plasma concentration of the free active substance may not be sufficient in the case of complex active substances and complex formulations. Indeed, even if plasmatic concentration profiles may be superimposable, the interaction and effect of nanomedicines with the plasmatic proteins and other human constituents may differ, with a potential impact on clearance, efficiency, and toxicity profiles.^[^ ^35^ ^]^ Consequently, the benefit/risk balance assessment of NBCDs and nanomedicine products should be supported by additional comparability studies able to demonstrate the similarity/equivalence of the two products in terms of quality, efficacy, and safety patterns. In the case of nanomedicine, the nanosystems play the main role in dictating the fate of the drug being involved in all interactions with biostructures including proteins and cells, and biodistribution and accumulation in tissues and organs while, in the case of traditional pharmaceutical products, the formulation affects the bioavailability by dictating the drug release. Therefore, the evaluation of bioavailability by assessing the drug profile in the blood cannot be the same in the case of nanotechnological and traditional formulations. In this context, both FDA and EMA have released guidelines on distinct types of nanomedicines (e.g., liposomes, iron‐core particles, and block‐copolymer‐micelle) to identify which additional data should be provided for characterizing first‐in‐human products or comparing follow‐on ones.^[^ ^7^, ^8^ ^]^ For the aforementioned class of nanomedicines, references are available in the literature to further deepen the discussion of critical quality attributes and other regulatory requirements for assessing quality, efficacy, and safety profiles.^[^ ^3^, ^34^ ^]^

However, some criticisms are still on the ground, affecting how healthcare authorities approach the problem of interchangeability (i.e., the possibility for a physician to indifferently opt for a specific medicine within a homogeneous class) and automatic substitution at the dispensing level (by a pharmacist) of such products in hospital and community settings. In this respect, it would seem appropriate for therapeutically equivalent copies of nanomedicine products to be evaluated by adopting regulatory principles closer to those used for the registration of biosimilars rather than generics.^[^ ^37^ ^]^

## 3 Quality Profile of Nanosystems: A Multidimensional Challenge

The physicochemical properties of a nanosystem mainly dictate its behavior in vivo. Indeed, the “crosstalk” between nanosystems and biological structures, including circulating macromolecules, cells, and tissues, occurs through surface interactions. The surface/size ratio of 3D systems results from the interplay of size/morphology/surface composition. The surface/volume ratio of 3D systems increases with the size decrease, while the surface/volume ratio depends on the morphology, having a minimal value in the case of spheres. Therefore, three parameters should be considered relevant for describing nanosystems’ physicochemical properties: a) particle size and polydispersity, b) morphology, and c) surface composition.^[^ ^35^ ^]^

Particle size is the main feature that identifies nanosystems and drives their behavior in vitro and in vivo, influencing the stability both in dispersion and physiological fluids, the penetration through tissues (including skin and mucosae), the biodistribution, and the elimination and, in turn, the pharmacokinetic (PK) profile.^[^ ^1^, ^38^, ^39^ ^]^ Considering the intravenous administration (excluding nanocrystals, most other nanomedicine products commercially available are approved for parenteral administration), the fate of a nanosystem depends, in the first instance, on the interaction with the blood proteins, which form the so‐called protein corona. Of note, the formation of such protein corona can drastically change the overall properties of nanosystems.

To distribute further into organs and tissues, nanosystems must escape from the immune system. The route of elimination depends on the diameter of a nanosystem: those with a size smaller than 10 nm are cleared by renal ultrafiltration whereas all larger structures are phagocytosed by liver and spleen macrophages and eliminated by the reticuloendothelial system (RES). The larger the system, the more efficient is this elimination process; nanosystems with an average diameter of 80–100 nm show the lowest RES macrophage uptake rate. In a work comparing the biodistribution of non‐PEGylated liposomes of different sizes (30–400 nm), it was demonstrated that liposomes smaller than 50 nm and larger than 250 nm are instead mostly cleared from circulation.^[^ ^40^ ^]^ Along with residence time in blood, also extravasation in diseased tissues (such as a tumor or inflamed tissues) based on enhanced permeability and retention effect is a size‐dependent phenomenon with nanosystems having a diameter in the range of the endothelium fenestrae (18–200 nm) that can accumulate in the target area exploiting the *Trojan horse* effect typical of nanosystems.

Along with particle size, morphology has a remarkable effect on both the clinical efficacy and safety of nanosystems. Indeed, on the one hand, the morphological properties of the surface dictate the biologically “active” area of the particles. On the other hand, surface characteristics determine particle movement through biological tissues and structures, including circulation in the bloodstream, tissue penetration, and cellular uptake. This is the case of Amphotericin B‐containing nanomedicines which were developed to minimize the severe renal toxicity induced by such antifungal drugs.^[^ ^41^ ^]^ Starting from the first authorized micellar formulation (Fungizone), lipidic nanosystems with ribbon‐like structures (Abelcet), disk‐like ones (Amphocil/Amphotec), and, lately, with a liposomal system (Ambisome), were successively introduced on the market. The pharmacokinetics and toxicology of these nanomedicines are strongly influenced by the morphology of the nanosystem. In fact, on the one side, ribbon‐like and disk‐like nanomedicines are characterized by a larger volume of distribution than that of liposomal formulations. On the other hand, however, although all formulations result in reduced renal toxicity, only the use of liposomal formulation displayed a lower incidence of infusion‐related side effects.

Another example of the impact of morphology, along with the surface charge, on the occurrence of hypersensitivity reactions to PEGylated nanosystems is the Doxil. Its well‐known immunological reactivity has been ascribed to the ovoidal shape of liposomes caused by the crystallization of doxorubicin in the aqueous core after the active loading by the ammonium sulphate gradient method.^[^ ^42^ ^]^ However, it is documented that equivalent formulations with a spherical shape did not lead to any increase in complement activation.

Surface properties are in turn the main responsible for the biological behavior of nanosystems in vivo. They are directly dictated by the composition of the surface and indirectly by particle size and shape. Surface charge, hydrophilic/hydrophobic balance, and specific targeting or coating moieties [e.g., poly(ethylene glycol), PEG] on the nanosystem surface result in their unspecific or specific interaction with biological components, such as mucus layers on mucosae, circulating proteins, and cells. Again, surface properties are strongly influenced by protein corona because absorbed proteins and macromolecules can lead to novel and different physical properties. This process is extremely complicated and still not well understood. However, grafting PEG onto a nanosystem surface is a well‐known formulation strategy to prevent protein binding, thereby prolonging blood circulation. On the other hand, the design of decorated nanosystems for active targeting or vaccines are designed increases the complexity of the manufacturing and quality‐control procedures. Indeed, the main regulatory authorities required a proper investigation of the chemical conjugation of the target/antigen moieties on the surface of the nanomedicine along with the study of its conformation which, in turn, influences the interaction with the receptors. Adding to this, surface modifications of a nanosystem can lead to changes in its toxicological pattern. For PEGylated nanosystems, published evidence based on spontaneous pharmacovigilance reports seems to suggest that drug‐related hypersensitivity reactions are triggered by PEGylation of the nanomedicine.^[^ ^43^ ^]^ Although further real‐world studies are needed, such findings corroborate how critical is to assess the quality profile of nanosystems, and the cause‐effect relationship between the quality critical attributes identified during the pharmaceutical development and the in vivo fate of the nanosystem.

### 3.1 Physicochemical Characterization of Nanomedicines

The characterization of nanomedicines in terms of particle size, morphology, and surface properties provides a minimum set of information for determining their quality pattern and predicting their interactions with biological constituents. This paragraph provides insights into the available techniques and the challenges for the physicochemical characterization of nanomedicine. An in‐depth discussion on methods applicable to evaluate, in vitro and in silico, the interaction between nanomedicines and biological constituents is available in refs. [44, 45].

The characterization of particle size and morphology of nanosystems is mandatory for all regulatory authorities^[^ ^5^ ^]^ and a plethora of methods are available to study these properties. These range from routine methods such as dynamic light scattering (DLS) and nanoparticle tracking analysis (NTA) to more complex techniques such as small angle X‐ray scattering (SAXS) and microscopy‐based techniques (i.e., transmission electron microscopy, TEM and cryogenic transmission electron microscopy, cryo‐TEM; atomic force microscopy, AFM), which provide also important structural information. It is noteworthy that, for some of these techniques, standards for the characterization of different nanotechnology‐based systems have been released or are under development by the International Organization for Standardization (ISO) or by the US National Cancer Institute Nanotechnology Characterization Laboratory (NCI‐NCL).^[^ ^46^, ^47^, ^48^, ^49^, ^50^, ^51^ ^]^ The most suitable characterization tool among those available should be selected depending on the type of nanosystem in the study. As an example, cryo‐TEM is an election method for studying the structure of liposomes, including liposomes‐micelles conversion phenomena, further complexations with chemical drugs and nucleic acids and it is also a powerful tool for the analysis of core–shell nanoparticles, but for instance, it is not a technique of choice in the case of polymeric matrixes such as poly(lactic‐co‐glycolic acid) (PLGA) nanoparticles. Moreover, mainly in the case of complex and polydisperse samples, the different analytical tools give complementary but not superimposable results. Focusing on the most routinely used methods, namely DLS and NTA, they both measure the hydrodynamic diameters of the dispersed particles according to the light scattering generated by the nanosystems under Brownian motion, but DLS measures an intensity particle size distribution, whereas NTA has the advantage to provide a number particle size distribution, allowing to discriminate better populations with close particle size and being less sensitive to the refractive index of the raw material.^[^ ^52^ ^]^ It is in line with the latest EC definition in which the nanomaterial is defined based on number size distribution. On the other hand, DLS allows to derive the surface charge of the nanosystems that affects both stability and interaction with the cells in vivo. Similarly, when a bimodal liposomal formulation was analyzed by DLS, cryo‐TEM, and multidetector asymmetric‐flow field flow fractionation (aF4), the last two techniques were able to distinguish two populations of vesicles having different shapes where DLS did not resolve the signal suggesting the presence of a monodisperse sample.^[^ ^53^ ^]^ It is a complicated but very versatile tool, suitable for the analysis of very polydisperse samples. aF4 is based on a parabolic laminar flow profile of the liquid mobile phase in a thin channel, without the need for a stationary phase, from which nanosystems of different sizes exit at different times, starting from the smallest size to the largest reaching multiple detectors such as UV‐spectrophotometer, refractive index, and DLS or multiangle static light scattering (MALS), which allow the measurement of particle size. The combination of MALS and DLS data provides the radius of gyration (*R*_g_) and hydrodynamic radius (*R*_h_), which can be used to calculate the shape factor parameter (*ρ* = *R*_g_/*R*_h_). It is worth noting that aF4 is also an election technique for the study of the stability of nanosystems in complex media such as blood and plasma because of the minimization of the interference of the protein fraction. This is an added value also in the study of the previously mentioned protein corona since this information is difficult to withdraw from routine data collected with the well‐known scattering techniques. The main issue related to this technique and in common with microscopy‐based ones is the need for highly qualified experts and the high analytical cost that make difficult their exploitation as routine quality control methods. For aF4, the reliability of the data strongly depends on the interpretation of the result whereas in the case of microscopy the sample preparation is critical because it is important, for example, to avoid the spreading of the nanosystems on the grid and/or substrate to keep unaltered the shape of the structures.

Characterizing the surface properties of nanosystems in general is not an easy task and requires using more sophisticated and often combined analytical methods. Among those, surface plasmon resonance and NMR are the most used methods to assess surface‐bound molecules.^[^ ^54^, ^55^ ^]^ X‐ray photoelectron spectroscopy and synchrotron small‐angle X‐ray scattering (SAXS) are other powerful tools to study nanosystems’ structure and surface properties. For example, SAXS allows obtaining especially useful information about PEG distribution and density of sterically stabilized nanosystems (i.e., liposomes or lipid nanoparticles in general).^[^ ^56^ ^]^ As for the case of cryo‐TEM and Atomic force microscopy, these tools require careful sample preparation and high competencies for data collection and interpretation to avoid misleading results.^[^ ^57^ ^]^ For further reading refers to a more detailed review of the methods of characterization of nanosystems.^[^ ^58^, ^59^ ^]^

### 3.2 Challenges in the Physicochemical Characterization of Nanomedicines

The peculiarities of the structural features, of the biorelevant media and/or biological systems used to test them, constitute the most relevant challenges in the characterization of nanosystems.^[^ ^18^ ^]^ The significance of the results obtained with the use of physicochemical methodologies should be weighed in terms of impact on nanosystem interactions with biological membranes. However, it is also important to stress that testing nanosystems' performances in biological models present additional challenges. Indeed, the features of biorelevant media and/or biological constituents (e.g., cells, tissue) may influence the biopharmaceutical performances of nanosystems. For example, it is known that for the same cell line or animal model, differences in phenotype can affect their ability to interact with nanosystems.^[^ ^44^ ^]^ As well, it is documented that small changes in the growing conditions of a cell line (e.g., growth substrate, incubation conditions, cell population) resulted in significant differences in their capacity to uptake the same nanosystems.

Both orthogonal approaches and harmonized protocols are needed to face such challenges. Indeed, on the one hand, the combination of different and more sophisticated analytical tools and the development/optimization of biorelevant biological models would improve the results both in terms of qualitative profile and biopharmaceutical relevance,^[^ ^45^, ^57^, ^60^ ^]^ although this requires the cooperation of scientists with diverse and highly specialized skills and knowledge. On the other hand, standardized protocols, specifically developed for pharmaceutical purposes, are essential from the characterization of the nanosystems’ performance in biorelevant media to in vitro studies on cell lines or ex vivo tissues.^[^ ^14^, ^44^, ^60^ ^]^ For example, regarding in vitro assays, such validated protocols should be detailed in terms of sample preparation and testing conditions (e.g., type of buffer, dilution of the sample, measurement setting) according to the quality target and the product profile of each class of nanomedicine and, consequently, for most critical quality attributes reported in the guidelines of regulatory authorities.^[^ ^3^, ^34^ ^]^ This would improve the repeatability and reproducibility of the results obtained across different laboratories, accelerating cooperation between industry and academic institutions. Furthermore, this would allow to establish robust and high‐quality databases on which to build AI/ML models to predict in silico the performance of nanosystems in vivo.^[^ ^14^ ^]^ Some attempts in such a direction are available worldwide. As documented by the latest guidance of the Joint Research Centre (JRC)^[^ ^61^ ^]^ and the available ISO standards,^[^ ^47^, ^48^, ^49^, ^50^, ^51^ ^]^ the EC efforts made in harmonized nanomaterials’ qualification have induced stakeholders to prioritize the standardization of so‐called “dry‐route” analytical methodologies (i.e., TEM, scanning electron microscopy (SEM), AFM), with respect to “dispersion route” methods (e.g., DLS, aF4). Indeed, based on the decision tree defined by JRC, the former should be privileged as confirmatory techniques for nanomaterials, while the latter should be used only for screening. However, such an approach presents evident weaknesses when translated to the pharmaceutical sector: a full characterization of the nanosystems dispersed in biorelevant media is crucial for predicting efficacy and safety patterns. In parallel, standards and Standard Operating Procedures (SOP) were developed by institutions with proven expertise in the physical, chemical, in vitro, and in vivo biological characterization of nanosystems for medical applications. It is the case of the efforts made by the European Nanomedicine Characterization Laboratory (EU‐NCL) and NCI‐NCL to develop SOPs for reliable orthogonal measurements of nanosystems with increasing complexity.^[^ ^46^ ^]^ The NCI‐NCL is established within the U.S. NCI with the express mission of accelerating the progress of nanomedicine by providing the preclinical characterization and safety testing of nanoparticles. Similarly, the EU‐NCL has been operating in Europe since 2019 as a consortium of eight European and one US institutions.^[^ ^62^ ^]^ The efforts of both NCI‐NCL and EU‐NCL in developing standardized analytical protocols are raising. For example, the NCI‐NCL makes available on the website several protocols on physicochemical characterization of nanosystems (e.g., size/size distribution, solution properties, surface chemistry, nanosystem concentration), immunological characterization (e.g., complement activation, phagocytosis), and other pharmacology and toxicology assessment (e.g., cytotoxicity, oxidative stress).

However, the important efforts made in the release of technical documents and reports have not yet translated into their widespread application within R&D laboratories. On the one hand, this may be because both the scientific and regulatory communities are not fully aware of all available information. On the other hand, the worldwide harmonization of characterization procedures is far from being defined as well‐advanced. The setup of experimental paraments or the analytical technique of choice may vary based on the source (e.g., NCI‐NCL, ISO, JRC) or geographic area of reference (e.g., EU vs US). Moreover, unlike what has been made for nanomaterials by the EC, there is still a lack of decision trees able to define and prioritize the characterization cascade that must be followed during the pharmaceutical development of a specific class of nanomedicines.

## 4 Conclusions and Future Perspectives

Due to their unique physicochemical characteristics and clinical behavior, nanomedicine products may represent a useful, efficient, and versatile therapeutic platform to develop personalized and optimized treatments. However, as mentioned above, several quality challenges and regulatory issues specific to nanomedicines are currently under critical discussion. In this regard, EMA and FDA have established recommendations for nanomedicine products,^[^ ^3^ ^]^ also creating an expert group to help achieve these regulatory goals.^[^ ^7^, ^8^ ^]^ As shown in Figure 1, the current challenges to facilitate the future development of nanomedicines can be identified in lack of: a) definitions applicable to pharmaceutical purposes, b) harmonized characterization approaches, c) in vitro biorelevant results that can predict the fate in vivo of nanomedicines, and d) sharing of updated knowledge between the scientific, industrial and regulatory communities.

To support biomedical R&D, regulatory agencies should continue to jointly implement their definitions of “nanotechnology” toward updated versions that take greater account of the peculiarities of the nanosystems for drug delivery and their quality target product profile. As discussed above, the available definitions appear qualitatively correct, but so extensive that they leave ample room for developers' interpretation. In this perspective, efforts should be made by the regulatory and scientific communities to reach a consensus on the univocal definition of nanomedicines through a more punctual specification of the criteria useful to better qualify nanotechnologies in their application to the pharmaceutical field. For example, in the case of the application of nanotechnologies to drug delivery, the updated definition should include general specifications for particle size polydispersity, morphology, and surface properties as well as other parameters relevant for more precise characterization of their biopharmaceutical properties.

Furthermore, despite the efforts of the scientific community, gaps still remain in terms of validated and standardized characterization methodologies for nanosystems.^[^ ^63^ ^]^ Existing guidelines from EMA and FDA have emphasized the need to provide a comprehensive characterization of nanosystems to identify critical quality attributes that may affect efficacy and safety profiles,^[^ ^7^, ^8^ ^]^ while private and scientific institutions are releasing protocols and procedures to support in vitro characterization of nanomedicines.^[^ ^11^, ^46^ ^]^ However, the current technical and regulatory framework is still far from having routine and standardized/compendial methods that can be used by pharmaceutical industries to assess the qualitative equivalence among batches of the same nanomedicine product. For example, it should be noted that, although the EMA guidelines have underlined the importance of fully characterizing the surface composition of PEGylated‐nanosystems,^[^ ^64^, ^65^ ^]^ such characterization should be based on the use of several highly sophisticated methodologies, using a harmonized multidisciplinary approach, to date not yet defined. Again, this limits the updating of Good Manufacturing Practices and the capability of regulators to provide specific and harmonized compliance and standards criteria for batch release laboratories of PEGylated nanomedicine products.

In this context, regulators could define regulatory decision trees (e.g., characterization cascades) and guidelines on analytical techniques and protocols that should be followed by manufacturers in order to obtain robust, biorelevant and comparable results in the characterization of similarity between nanosystems containing the same active principle. At least, they should recommend methodologies capable of determining particle size, polydispersity, morphology, and surface composition (particularly applicable to decorated nanosystems). Furthermore, such flowcharts should also distinguish between methods and protocols that could be used in routine quality controls and those that are more appropriate for the pharmaceutical development of first‐in‐human products and/or comparability studies for copies/follow‐on products. For the latter, the equivalence of complex nanomedicines poses significant scientific, medical, and regulatory challenges in providing evidence of sufficient similarity. In this regard, criteria have been proposed to select and characterize nanosimilars.^[^ ^32^ ^]^ These include at least: particle size and size distribution, particle surface characteristics, the fraction of uncaptured bioactive moiety, stability on storage, bioactive moiety uptake and distribution, and stability for ready‐to‐use preparations. Moreover, a quality comparison should also include the assessment of the stability of follow‐on nanomedicines in plasma and the characterization of its protein corona pattern with respect to the originator nanomedicine. In addition to that, in vitro cell culture studies may be required based on the class of nanomedicine products and intended therapeutic indications.

It is worth noting that while such initiatives are per se strategic for the development of next‐generation nanomedicines, they should be carried out in an integrated way between academic or industrial scientists and regulatory experts. It is indeed mandatory to promote a structural interaction between the scientific, industrial, and regulatory communities to support sharing of knowledge and, therefore, to accelerate the harmonization and standardization of multilevel procedures. Obviously, this process passes through international recognition and the consolidation of educational and scientific networks between scientific institutions, regulatory agencies, and analytical facilities operating in the characterization of nanotechnologies at a global level (e.g., EU‐NCL project^[^ ^62^ ^]^).

Although such solutions would be sufficient for “established” nanomedicines and their follow‐on products, the discovery and development of more sophisticated nanosystems (e.g., RNA‐based nanomedicine) will lead to additional challenges in fully understanding their pharmacokinetic and pharmacodynamic properties, or their toxicological profiles.^[^ ^35^, ^66^ ^]^ This underlines the need to enhance the efforts of academics, clinicians, manufacturers, and policymakers to better understand the relationship between physicochemical properties and the efficacy and safety patterns of nanomedicines. Such an objective should be pursued through the implementation of in vitro assays and in vitro/in vivo correlation models by applying AI/ML tools.^[^ ^60^, ^67^ ^]^ However, in this context, all the challenges mentioned above (e.g., harmonized definitions and standardization of analysis protocols) appear even more strategic. The robustness and relevance of AI/ML‐generated models are greatly influenced by the availability of large databases of data generated on adequately defined nanosystems under standardized conditions.^[^ ^14^ ^]^ Meanwhile, such a lack of knowledge about nanomedicines makes it difficult to formulate reliable guidelines and create adequate regulations that can reduce patient safety to zero. Thankfully, the massive digitalization of Western societies is opening new and innovative solutions (e.g., blockchain technologies, multimodal computational systems) to minimize patient risks, enabling potential real‐time collection of use‐related adverse effects of nanomedicine products.^[^ ^13^ ^]^

In the future, to address the challenges arising from the commercialization of an increasing number of nanomedicines, an integrated and unifying approach for their assessment should be developed and shared among academic institutions, industrial stakeholders, and regulatory authorities. This means investing in the development of more biorelevant in vitro assays, in the standardization of qualification and characterization procedures, in tuning robust in vitro/in vivo correlation models, and in the digitalization of both research and postmarketing vigilance to obtain a more reliable understanding of the impact of nanomedicines’ features on their fate in vivo. The resulting release of joint harmonized guidelines for the development and evaluation will improve risk assessment of the use of nanomedicines, enhancing the legal certainty of regulatory pathways for approval of such medicine products.

## Conflict of Interest

The authors declare no conflict of interest.
